# Performance de l´imagerie par résonance magnétique dans l´approche histopathologique des tumeurs parotidiennes

**DOI:** 10.11604/pamj.2021.39.10.27813

**Published:** 2021-05-04

**Authors:** Mohamed Masmoudi, Mehdi Hasnaoui, Rihab Guizani, Rihab Lahmar, Saida Jerbi, Khalifa Mighri

**Affiliations:** 1 Département d´Oto-rhino-laryngologie, Hôpital Tahar Sfar Mahdia, Mahdia, Tunisia,; 2 Département d´Imagerie Médicale, Hôpital Tahar Sfar Mahdia, Mahdia, Tunisia

**Keywords:** Tumeur, glande parotide, IRM, cancer, Warthin, adénome pléomorphe, Tumor, parotid gland, MRI, cancer, Warthin, pleomorphic adenoma

## Abstract

**Introduction:**

la glande parotide est le siège préférentiel des tumeurs des glandes salivaires. Ces tumeurs sont rares mais se distinguent par une grande diversité histologique, posant ainsi des difficultés diagnostiques. L´imagerie par résonance magnétique (IRM) constitue actuellement le moyen d´imagerie le plus fiable dans l´exploration de ces tumeurs. Le but de notre étude était de préciser la valeur diagnostique de l'IRM et son rôle dans l'approche histopathologique de ces tumeurs.

**Méthodes:**

il s´agissait d´une étude rétrospective descriptive et analytique ayant porté sur 50 cas de tumeurs de la parotide, opérés et pris en charge dans le service d´oto-rhino-laryngologie, et de chirurgie cervico-faciale de l´Hôpital Taher Sfar de Mahdia entre 2001 et 2019. Tous nos patients ont été explorés par une IRM parotidienne en préopératoire.

**Résultats:**

parmi les 50 patients inclus dans notre étude, 36 (72%) présentaient une tumeur bénigne et 14 (28%) une tumeur maligne. La sensibilité de l´IRM pour le diagnostic de malignité était de 92,8% avec une spécificité de 97,2%, une valeur prédictive négative de 93% et une valeur prédictive positive de 97%. Concernant la caractérisation des tumeurs bénignes, l´IRM a évoqué le diagnostic de tumeur de Warthin dans tous les cas (13 cas) et le diagnostic d´adénome pléomorphe dans 22 cas parmi 23. Il y´avait 2 erreurs diagnostiqués: l´IRM a évoqué à tort le diagnostic d´adénome pléomorphe dans un cas de carcinome adénoïde kystique et elle a évoqué une tumeur maligne dans un cas d´adénome pléomorphe devant une restriction de diffusion.

**Conclusion:**

l´IRM est hautement performante dans l´appréciation de la nature histologique de la tumeur parotidienne et surtout après l´avènement des nouvelles séquences fonctionnelles. Toutefois, seule l´analyse histologique permet de confirmer avec certitude le diagnostic de nature de la lésion.

## Abstract (en)

**Introduction:**

salivary gland tumors mainly occur in the parotid gland. These tumors are rare but are characterized by histological heterogeneity, thus posing diagnostic challenges. Magnetic resonance imaging (MRI) is currently the most reliable imaging test for the evaluation of these tumors. The purpose of this study was to highlight the diagnostic value of MRI and its role in parotid gland tumor histopathology.

**Methods:**

we conducted a retrospective descriptive and analytical study of 50 patients with parotid gland tumor, operated and treated in the ear, nose and throat (ENT) Department and in the Department of cervicofacial surgery at the Tahar Sfar University Hospital of Mahdia between 2001 and 2019. All patients underwent preoperative MRI of the parotid gland.

**Results:**

out of 50 patients included in the study, 36 (72%) had benign tumor and 14 (28%) malignant tumor. The sensitivity of MRI for the diagnosis of malignant tumor was 92.8% with a specificity of 97.2%, a negative predictive value of 93% and a positive predictive value of 97%. With respect to benign tumor characterization, MRI suggested the diagnosis of Warthin tumor in all cases (13 cases) and of pleomorphic adenoma in 22 out of 23 cases. There were two diagnostic errors: MRI suggested the diagnosis of pleomorphic adenoma instead of adenoid cystic carcinoma in one case and of malignant tumor instead of pleomorphic adenoma due to diffusion restriction.

**Conclusion:**

MRI is highly efficient in the assessment of parotid tumor histology and, especially, after the advent of new functional sequences. However, only histological examination allows to confirm with certainty the diagnosis.

## Introduction

Les tumeurs des glandes salivaires constituent une entité relativement rare, avec une incidence annuelle de 0,4 à 13,5 cas pour 100 000 habitants [1]. Ces tumeurs ont une localisation essentiellement parotidienne (80%) et sont caractérisées par une grande diversité histologique, pouvant parfois poser des difficultés diagnostiques [1-3]. L´IRM constitue actuellement le moyen d´imagerie le plus fiable dans l´exploration des tumeurs parotidiennes. Elle permet d´établir le diagnostic positif, fournir un bilan d´extension locorégionale et offrir une approche histologique de bénignité ou de malignité [4].

## Méthodes

Il s´agit d´une étude rétrospective sur 50 cas de tumeurs parotidiennes opérés et pris en charge dans notre service entre 2001 et 2019 soit sur une période de 19 ans. Tous nos patients ont été explorés par une IRM parotidienne réalisée sur une machine General Electrique de 1.5 tesla. Les séquences conventionnelles (une séquence en pondération T1 + une séquence en pondération T2 sans saturation du signal de la graisse + une séquence T1 avec suppression de la graisse et injection de Gadolinium) ont été réalisées dans tous les cas et les séquences fonctionnelles ont été réalisées dans 27 cas pour les séquences de diffusion et dans 21 cas pour les séquences de perfusion. Toutes les IRMs ont eu une relecture par un expert en imagerie parotidienne.

L´étude statistique a été descriptive puis analytique à la recherche d´une relation entre un type histologique donné d´une tumeur parotidienne et ses différentes caractéristiques à l´IRM. L´étude analytique était uni et multivariée: l´étude univariée étudie la relation entre le type histologique de la tumeur (adénome pléomorphe, tumeur de warthin, tumeur maligne) et les caractéristiques épidémiologiques, cliniques et paracliniques. Toutes les variables étaient qualitatives. Les tests statistiques utilisés étaient le test de Chi2 de Pearson si l´effectif des sous-groupes est > 5 et le test exact de Fisher si l´effectif des sous-groupes est < 5. Pour l´étude multivariée, on a sélectionné les variables déclarées en relations significatives avec chaque type histologique lors de l´étude univariée puis on les a soumis à une régression logistique afin d´éliminer toute interdépendance entre les variables. Pour toutes nos analyses statistiques, le seuil de signification a été fixé à 5%.

Ensuite, le diagnostic proposé par l´IRM (en termes de nature maligne/bénigne de la tumeur) a été comparé avec le diagnostic histopathologique définitif. Pour cette modalité diagnostique, nous avons calculé la sensibilité, la spécificité, la valeur prédictive négative (VPN) et la valeur prédictive positive (VPP).

## Résultats

Parmi les 50 tumeurs parotidiennes colligées, nous avons dénombré: trente-six tumeurs bénignes (72%) et 14 tumeurs malignes (28%). L´adénome pléomorphe était le type histologique le plus fréquent, observé dans 23 cas (46%), suivi par la tumeur de Warthin qui était notée dans 13 cas (26%) (Tableau 1). L´âge moyen de nos patients était de 42 ans avec des extrêmes de 16 et 75 ans. Une discrète prédominance masculine était notée, il s'agissait de 30 hommes (60%) et 20 femmes (40%) soit un sex-ratio de 1.5. Le motif de consultation était une tuméfaction parotidienne dans 100% des cas. Cette tuméfaction était douloureuse dans 10 cas (20%), dure dans 9 cas (18%) et fixe dans 6 cas (12%). Une paralysie faciale périphérique (PFP) a été objectivée chez 4 patients et des adénopathies (ADPs) cervicales ont été retrouvées dans 3cas.

**Tableau 1:** fréquence des tumeurs parotidiennes bénignes et malignes

Caractéristiques de la tumeur à l´IRM		Tumeurs bénignes (36 cas)	Tumeurs malignes (14 cas)
**Séquences conventionnelles**	**Contours**	Bien définis 36 cas (100% des tumeurs bénignes)	Mal définis 7 cas (50% des tumeurs malignes)
	**Signal**	Hyposignal T1 (100%) Zones en hyper T1: 14 (38,8%) Hypersignal T2: 21 cas (58,3%) Isosignal T2: 12 (27,7%) Hyposignal T2: 3 cas (11,1%)	Hyposignal T1 (100%) Zones hyper T1: 1cas (7,1%) Hyersignal T2: 5 cas (35,7%) Isosignal T2: 1 cas (7,1%) Hyposignal T2: 8 cas (57%)
	**Envahissement des structures de voisinage -**		7 cas (50%)
	**Infiltration péri-neurale**		-
	**Adénopathies cervicales -**		3 cas (21,4%)
**Séquences fonctionnelles**	**Courbe dynamique de rehaussement: 21 cas**	Type A ou B: les 13 lésions bénignes étudiés (100%)	Type C: les 8 cas étudiés (100%)
	**Diffusion (rADC): 27 cas**	ADC augmentée (rADC >1): 12 cas parmi 19 lésions bénignes étudiées (63,1%)	Restriction d´ADC (rADC<1): 8 cas étudiés (100%)

ADC: coefficient de diffusion; rADC: rapport de coefficient de diffusion (ADC tumeur/ADC parotide saine)

L´IRM a permis de différencier entre tumeur bénigne et maligne grâce à différents critères (Tableau 2): l´aspect des contours, le signal de la lésion notamment en T2, la présence ou non d´envahissement des structures de voisinage et des adénopathies suspectes. L´envahissement péri-neural (particulièrement observée dans le carcinome adénoïde kystique) n´a été noté dans aucun cas de tumeurs malignes. Les séquences fonctionnelles ont permis de distinguer entre tumeur bénigne et maligne en se basant sur l´aspect de la courbe dynamique de rehaussement et le rapport de coefficient de diffusion (rADC) comparativement au parenchyme sain (ADC tumeur/ADC parotide saine). Dans notre étude, les critères significativement liés aux tumeurs malignes étaient: le caractère mal limité (p = 0,0001), l'aspect en hyposignal T2 de la lésion (p = 0,001), la présence d´adénopathies suspectes (0,004), l'envahissement locorégional (p = 0,035), une courbe dynamique de type C (p = 0,001). En revanche, nous n´avons pas retrouvé de différence significative entre les tumeurs bénignes et malignes en ce qui concerne les valeurs d´ADC (p = 0,17).

**Tableau 2:** récapitulatif des caractéristiques des tumeurs parotidiennes à l´IRM

	Type histologique	Nombre	Pourcentage /ensemble des tumeurs
**Tumeurs bénignes** 36 cas (72%)	Adénome pléomorphe	**23**	**46%**
	Tumeur de Warthin	13	**26%**
**Tumeurs malignes** 14 cas (28%)	Carcinome adénoïde kystique	**3**	6%
	Carcinome ex- adénome pléomorphe	2	4%
	Carcinome muco-épidermoïde	2	4%
	Adénocarcinome SAI	2	4%
	Carcinome à cellules acineuses	2	4%
	Tératome immature	1	2%
	Lymphome type MALT	1	2%
	Carcinome épidermoïde	1	2%

Concernant les tumeurs bénignes, la majorité des adénomes pléomorphes (91,3%) présentait un hypersignal intense en T2 (p = 0,0002), avec une capsule en hyposignal T2 dans 87% des cas (p = 0,002) et des contours lobulés dans 95,7% des cas (p = 0,001). Une étude dynamique a été réalisée dans 8 cas montrant : une courbe dynamique de type A dans tous les cas (p = 0,001) avec un rADC >1,3 dans 76,9% des cas (p=0,01). Par ailleurs, la majorité des tumeurs de Warthin (84,6%) était localisée au niveau du pôle inférieur de la glande (P = 0,0001). Des zones d´hypersignal T1 étaient notées dans tous les cas (P = 0,001). L´étude dynamique était faite seulement dans 5 cas. Tous avaient une courbe dynamique de type B (p = 0,003) et un ADC inférieur à 1 (p = 0,002). En analyse multivariée, les caractéristiques significativement liées aux tumeurs malignes, à l´AP et à la TW étaient respectivement: l´aspect mal limité de la lésion pour les tumeurs malignes (p = 0,001), l´aspect en hypersignal T2 franc pour l´AP (p = 0,00004) et la présence des spots en hypersignal T1 pour la TW (p = 0,0001).

**Figure 1:**
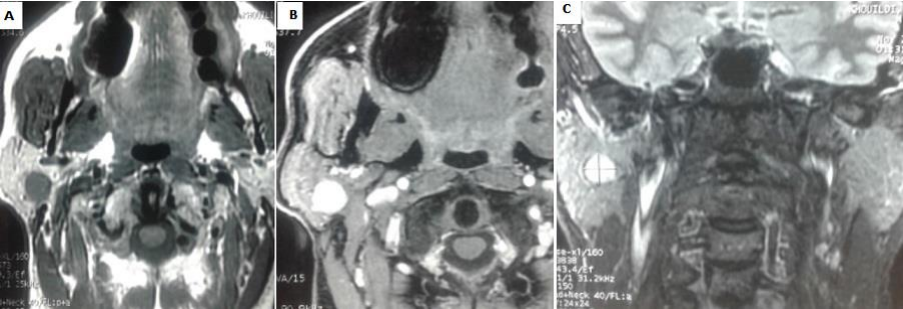
IRM parotidienne d´un cas de carcinome adénoïde kystique en coupes axiales T1 (A), T1 FAT SAT Gado (B) et coronale T2 (C): lésion parotidienne droite bien limitée en hyposignal T1 et hypersignal T2 qui se rehausse de façon intense et homogène après injection de Gadolinium

**Figure 2:**
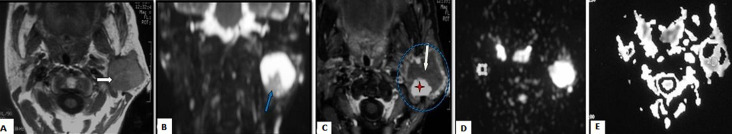
IRM parotidienne d´un cas d´adénome pléomorphe kystisé en coupes axiale T1 (A), coronales T2 FAT SAT (B) et T1 FAT SAT GADO (C) diffusion (D) et cartographie ADC (E): (A)+(B): masse ovalaire de contour régulier du lobe superficiel de la parotide gauche (flèche blanche) en hypo signal T1, hyper signal T2 hétérogène (remaniement kystique) (c) + (d) + (e): un rehaussement intense et homogène de la composante charnue (étoile) avec une large composante kystique (flèche) en hyper signal hétérogène sur la séquence de diffusion avec restriction d´ADC (rADC = 0,62) de la composante charnue (étoile)

Après comparaison des données de l´imagerie aux données histologiques définitives, nous avons remarqué que la sensibilité de l´IRM pour le diagnostic de malignité était de 92,8% avec une spécificité de 97,2%, une VPP de 93% et une VPN 97%. Par ailleurs il y´avait deux erreurs diagnostiqués: dans le premier cas, l´IRM a évoqué à tort le diagnostic d´adénome pléomorphe en se basant seulement sur l´étude morphologique alors que l´examen histologique définitif a conclu à un carcinome adénoïde kystique (Figure 1). Pour le deuxième cas, l´RM a évoqué une tumeur maligne devant la restriction de diffusion de la composante charnue avec un rADC bas à 0,62 dans un cas d´adénome pléomorphe kystisé (Figure 2). Concernant la caractérisation des tumeurs bénignes, l´IRM a évoqué le diagnostic de tumeur de Warthin dans tous les cas (13 cas) et le diagnostic d´adénome pléomorphe dans 22 cas parmi 23.

## Discussion

Les tumeurs parotidiennes représentent 3% des tumeurs cervicales et 80% des tumeurs salivaires. Elles se caractérisent par une grande diversité morphologique et histologique, mais ce sont les formes bénignes qui prédominent (80% des cas) avec en chef de file l´adénome pléomorphe [1, 2]. Les tumeurs malignes représentent 10 à 15% des tumeurs parotidiennes et sont dominées par le carcinome muco-épidermoïde [2, 3]. Le mode de révélation le plus fréquent est une tuméfaction de la loge parotidienne [1]. L´approche diagnostique face à une tumeur de la parotide se base sur le trépied fondamental: la clinique, l´imagerie et la cytoponction à l´aiguille fine [1, 2, 4]. L´intérêt de l´imagerie est de procurer au clinicien une information anatomique ainsi qu´une information sur la nature de la tumeur investiguée. L´IRM représente un examen non invasif, non irradiant, permettant la réalisation de coupes dans des plans multiples [5]. Elle doit comprendre des séquences conventionnelles et d´autres fonctionnelles [6-8].

Les séquences conventionnelles offrent une étude morphologique avec un bilan d´extension locorégional précis. Celles-ci permettent aussi de rechercher un éventuel envahissement péri neural particulièrement observé dans le cas des carcinomes adénoïdes kystiques. En contrepartie, les séquences fonctionnelles (de perfusion et de diffusion) sont plus performantes que les séquences conventionnelles dans la caractérisation de la matrice tumorale et la différenciation entre tumeurs bénignes et malignes [6, 8, 9]. L´IRM est hautement performante dans l´appréciation du caractère bénin ou malin de la tumeur et dans le typage histologique des tumeurs bénignes. Par ailleurs, l'étude des principales séries publiées fait apparaître que la sensibilité de l´IRM était variable de 80 à 93,3%, et que sa spécificité variait de 85 à 100% [6, 8, 10-12]. Dans notre étude, la sensibilité et la spécificité étaient respectivement de 92,8% et 97,2%, proches de celles de la littérature. L´IRM permet de prévoir la malignité d´une tuméfaction parotidienne grâce à différents critères: l´aspect mal limité des contours, l´hyposignal T2 de la lésion, l´infiltration péri-lésionnelle et péri-neurale (particulièrement observée dans le carcinome adénoïde kystique) et la présence d´adénopathie suspecte. Aucun de ces critères pris seul n´est pathognomonique et c´est l´association de 4 critères ou plus qui oriente vers le diagnostic de malignité [1, 7].

Cependant, certaines lésions restent indéterminées sur les seuls critères morphologiques tel que le carcinome adénoïde kystique qui peut être en hypersignal T2 et être donc difficilement différencié d´un adénome pléomorphe ou être le siège d´une nécrose hémorragique à l´origine d´hypersignaux T1, mimant ainsi une tumeur de Warthin. Dans ces cas, les séquences fonctionnelles sont indispensables car elles vont rechercher des arguments supplémentaires en faveur de la malignité [9, 13, 14]. Dans notre série, en se basant uniquement sur les données de l´étude morphologique, l´IRM a évoqué à tort le diagnostic d´adénome pléomorphe dans un cas de carcinome adénoïde kystique. D´où l´intérêt des séquences fonctionnelles qui n´ont pas été réalisées dans ce cas.

Classiquement, les tumeurs malignes ont un rADC assez bas en raison de leur hypercellularité. La plupart des études retrouvent une différence significative entre le rADC des lésions bénignes et celui des lésions malignes [7, 14]. En revanche, dans notre étude, nous n´avons pas retrouvé de différence significative entre les tumeurs bénignes et malignes en ce qui concerne les valeurs de rADC (P = 0,17). Ceci peut être expliqué par le fait qu´il existe un chevauchement considérable entre les valeurs de rADC des différents groupes de tumeurs bénignes et malignes [13]. Sur la séquence de perfusion, les tumeurs malignes présentent classiquement une courbe de type C (c'est-à-dire un pic précoce sans lavage au temps tardif) [8, 9]. En ce qui concerne la caractérisation des lésions bénignes, l´adénome pléomorphe se présente à l´IRM [7, 12] comme une lésion bien limitée, avec des contours lobulés, en hyposignal T1, hypersignal T2 franc et qui se rehausse généralement de façon homogène après injection de produit de contraste. Souvent l´aspect lobulé n´est retrouvé que dans la moitié des cas et son signal en T2 peut être hétérogène. En diffusion, la tumeur apparait hyper intense et le rADC est augmenté (supérieur à 1,3). La courbe de type A est très majoritairement rencontrée.

Dans notre étude, la description IRM de l´adénome pléomorphe répondait en grande partie aux descriptions de la littérature. Le diagnostic d´adénome pléomorphe à l´IRM, était exact dans 22 cas parmi 23 (95,6% des cas). En effet la malignité était suspectée à tort dans un cas d´adénome pléomorphe kystisé devant un rADC bas (0,62). Cette valeur non concordante avec la littérature est expliquée par le mal positionnement de la région d´intérêt (ROI) au moment de calcul de rADC. Par ailleurs la tumeur de Warthin est perçue à l´IRM comme une lésion bien limitée, intéressant communément la partie superficielle du pôle inférieur de la glande et ayant un signal intermédiaire en T1 et T2. Les zones en hypoT2 hyper T1 correspondent à des remaniements hémorragiques ou des portions kystiques riches en cristaux de cholestérol [6, 7]. Le rADC se situe aux alentours de 1. Cette tumeur présente une courbe de type B qui s´explique par le compte élevé de micro-vaisseaux et la forte cellularité du stroma [8].

Au total, en se basant sur les résultats de l´étude multivariée de notre série les tumeurs parotidiennes avaient pour signes pathognomoniques à l´IRM: l´hypersignal T2 franc pour l´adénome pléomorphe (P = 0,0004), la présence de spots en hypersignal en T1 pour la tumeur de Warthin (p = 0,0001) et la présence de contours mal définis pour les tumeurs malignes (P = 0,001). Ces résultats sont en accord avec les données de la littérature.

Le caractère mal limité de la lésion à l´IRM, était le signe le plus pourvoyeur de malignité avec une efficacité diagnostique de 95% et une sensibilité de 81,2%. L´aspect en hyposignal T2 de la lésion, était le plus souvent corrélé à un haut grade histologique et ne permettait pas de faire la distinction entre les tumeurs malignes, les tumeurs de Warthin et les oncocytomes [1, 4, 10, 12].

Par ailleurs, l´IRM réalisée théoriquement avant la cytoponction (pour écarter tous les phénomènes de remaniements hémorragiques qu'elle engendre) garde une sensibilité meilleure que celle de la cytoponction pour le diagnostic des carcinomes de bas grade et des lymphomes [11, 13, 15]. En effet, les erreurs diagnostiques en cytologie sont fréquentes dans le cas des tumeurs malignes de bas grade, les lymphomes et dans les lésions kystiques pauci-cellulaires [15-17]. D´où une stratégie diagnostique combinée cytoponction/IRM serait toujours intéressante [9, 14].

## Conclusion

L´IRM est actuellement l´examen d'imagerie le plus performant, le plus reproductible et le plus valide dans la stratégie d´investigation préopératoire des tumeurs parotidiennes. Elle fournit une étude précise des caractéristiques de la tumeur et de l´environnement péri-tumoral. Elle est hautement performante dans l´appréciation du caractère bénin ou malin et dans le typage histologique des tumeurs bénignes surtout après l´avènement des nouvelles séquences dynamiques (de diffusion et de perfusion). Toutefois, seule l´analyse histologique permet de confirmer avec certitude le diagnostic de nature de la lésion.

### Etat des connaissances sur le sujet

- Jusqu´à nos jours, l´évaluation précise préopératoire des tumeurs parotidiennes demeure un véritable challenge pour les cliniciens, les radiologues et les cytopathologistes pour prédire la nature de la lésion, en sachant que c´est de la nature histologique de la tumeur que découlera la planification de l´acte chirurgical avec les conséquences et les complications qui en résulteront;
- L´IRM constitue actuellement le moyen d´imagerie le plus fiable dans l´exploration des tumeurs de la parotide; toutefois, l'efficacité de cet examen dans l´approche de la nature histologique exacte de la tumeur n'est pas encore bien définie.

### Contribution de notre étude à la connaissance

- Au terme de notre étude nous sommes arrivés à conclure à une sensibilité et une spécificité à l'IRM de 92,8% et 97,2% respectivement;
- Il y´avait deux erreurs diagnostiques de l´IRM dont l´un est dû à la réalisation uniquement des séquences conventionnelles;
- D'où la nécessité d´associer systématiquement les nouvelles séquences fonctionnelles de l'IRM (diffusion et perfusion) à ces séquences classiques puisqu´elles ont démontré des résultats prometteurs en matière de différenciation entre tumeur maligne et bénigne, et aussi dans la caractérisation de certaines tumeurs bénignes (tel que l´adénome pléomorphe et la tumeur de Warthin).

## References

[1] Bouaity B, Darouassi Y, Chihani M, Touati MM, Ammar H (2016). Les facteurs prédictifs de malignité dans la prise en charge des tumeurs parotidiennes: à propos de 76 cas. Pan Afr Med J.

[2] Maahs GS, Oppermann P de O, Maahs LGP, Machado Filho G, Ronchi AD (2015). Parotid gland tumors: a retrospective study of 154 patients. Braz J Otorhinolaryngol.

[3] Kennedy RA (2018). WHO is in and WHO is out of the mouth, salivary glands, and jaws sections of the 4^th^ edition of the WHO classification of head and neck tumours. Br J Oral Maxillofac Surg.

[4] Thielker J, Grosheva M, Ihrler S, Wittig A, Guntinas-Lichius O (2018). Contemporary Management of Benign and Malignant Parotid Tumors. Front Surg.

[5] Cicero G, D´angelo T, Racchiusa S, Salamone I, Visalli C, Bottari A (2018). Cross-sectional Imaging of Parotid Gland Nodules: A Brief Practical Guide. J Clin Imaging Sci.

[6] Pommier A, Lerat J, Orsel S, Bessede J-P, Aubry K (2017). Corrélation cyto-histologique dans la prise en charge des tumeurs parotidiennes : étude rétrospective de 160 cas. Bulletin du Cancer.

[7] Espinoza S, Halimi P (2013). Les éléments clés de l´interprétation de l´IRM des tumeurs parotidiennes. Annales françaises d´Oto-rhino-laryngologie et de Pathologie Cervico-faciale.

[8] Mikaszewski B, Markiet K, Smugała A, Stodulski D, Szurowska E, Stankiewicz C (2017). Diffusion-and Perfusion-Weighted Magnetic Resonance Imaging-An Alternative to Fine Needle Biopsy or Only an Adjunct Test in Preoperative Differential Diagnostics of Malignant and Benign Parotid Tumors?. J Oral Maxillofac Surg. oct.

[9] Elkind L (2016). Apport des séquences IRM de diffusion et de perfusion dans la caractérisation des tumeurs de la parotide : étude prospective de 42 lésions.

[10] Zouhair N, Mallouk S, Oukessou Y, Rouadi S, Abada RL, Roubal M (2020). Corrélation entre l´imagerie par résonnance magnétique, l´extemporanée et l´histologie définitive des tumeurs parotidiennes: série de cas. Pan Afr Med J.

[11] Wang J, Takashima S, Takayama F, Kawakami S, Saito A, Matsushita T (2001). Head and neck lesions: characterization with diffusion-weighted echo-planar MR imaging. Radiology.

[12] Padhani AR, Liu G, Koh DM, Chenevert TL, Thoeny HC, Takahara T (2009). Diffusion-weighted magnetic resonance imaging as a cancer biomarker: consensus and recommendations. Neoplasia.

[13] Garrett SL, Trott K, Sebastiano C, Wolf MJ, Rao NK, Curry JM (2019). Sensitivity of Fine-Needle Aspiration and Imaging Modalities in the Diagnosis of Low-Grade Mucoepidermoid Carcinoma of the Parotid Gland. Ann Otol Rhinol Laryngol.

[14] Fassih M, Abada R, Rouadi S, Mahtar M, Roubal M, Essaadi M (2014). Les tumeurs des glandes salivaires, étude épidémio-clinique et corrélation anatomoradiologique: étude rétrospective à propos de 148 cas. Pan Afr Med J.

[15] Gillespie MB, Walvekar RR, Schaitkin BM, Eisele DW (2018). Gland-Preserving Salivary Surgery: A Problem-Based Approach.

[16] Park YM, Oh KH, Cho J-G, Baek S-K, Kwon S-Y, Jung K-Y (2018). Analysis of efficacy and safety of core-needle biopsy versus fine-needle aspiration cytology in patients with cervical lymphadenopathy and salivary gland tumour. Int J Oral Maxillofac Surg.

[17] Gudmundsson JK, Ajan A, Abtahi J (2016). The accuracy of fine-needle aspiration cytology for diagnosis of parotid gland masses: a clinicopathological study of 114 patients. J Appl Oral Sci.

